# Polypharmacy in HIV: Rethinking what counts and why it matters

**DOI:** 10.1111/hiv.70129

**Published:** 2025-10-21

**Authors:** Luxsena Sukumaran, Alan Winston, Catia Marzolini, Saye Khoo, Marta Boffito, Nadia Naous, Caroline A. Sabin

**Affiliations:** ^1^ Institute for Global Health University College London London UK; ^2^ National Institute for Health and Care Research (NIHR) Health Protection Research Unit (HPRU) in Blood‐borne and Sexually Transmitted Infections at University College London London UK; ^3^ Department of Infectious Disease, Imperial College London London UK; ^4^ Service of Clinical Pharmacology, Department of Laboratory Medicine and Pathology, University Hospital Lausanne University of Lausanne Lausanne Switzerland; ^5^ Division of Infectious Diseases, University Hospital Basel University of Basel Basel Switzerland; ^6^ Centre for Experimental Therapeutics University of Liverpool Liverpool UK; ^7^ Chelsea and Westminster Healthcare NHS Foundation Trust London UK

**Keywords:** deprescribing, HIV, medication burden, polypharmacy, review

## Abstract

Polypharmacy, the concurrent use of multiple medications, presents a growing challenge in HIV care as people living with HIV age and experience earlier onset of age‐related co‐morbidities. However, how polypharmacy is defined and assessed in HIV research remains inconsistent. The commonly used threshold of five or more medications, often derived from geriatric medicine, may not adequately reflect the clinical complexity of HIV care, where lifelong antiretroviral therapy (ART) forms the foundation of treatment. This review examines how polypharmacy has been defined and operationalized in HIV studies and compares this to approaches in geriatric research, where tools (e.g., STOPP/START and the Beers criteria) have been more systematically applied. We argue that HIV care can benefit from, but must also adapt, these frameworks to address the unique pharmacologic, psychosocial and adherence‐related considerations faced by people with HIV. We also review emerging evidence linking polypharmacy in HIV with negative outcomes, including increased risk of drug–drug interactions, hospitalization, reduced quality of life, and associated healthcare costs. At the same time, polypharmacy is not inherently inappropriate, as many regimens may reflect guideline‐concordant care. Rather than focusing on medication count alone, attention should shift toward evaluating appropriateness, safety and alignment with the individual's evolving health needs. Finally, we explore the role of deprescribing in HIV care, acknowledging both its promise and the challenges it presents, particularly in preserving ART stability and supporting shared decision‐making. Reframing polypharmacy through an HIV‐specific lens can support safer prescribing and improve outcomes as the HIV population continues to age.

## INTRODUCTION

As the global population of people with HIV ages, clinical priorities have shifted from virological control, which is generally achievable, to managing long‐term issues such as multimorbidity and polypharmacy [1, 2], defined as the concurrent use of multiple medications [3]. In addition to their antiretroviral therapy (ART) medications, an estimated 25%–42% of people living with HIV concurrently take multiple non‐ART medications, with prevalence rising from 35% to 94% among those aged ≥50 years [4]. In older populations without HIV, polypharmacy has been associated with negative outcomes, including adverse drug events, functional decline, and hospitalization [5, 6]. These findings have prompted the development of prescribing tools, such as the Screening Tool of Older People's Prescriptions/Screening Tool to Alert to Right Treatment (STOPP/START) criteria [7] and Beers criteria [8]. Although insights from geriatric care offer a useful foundation, they may not fully capture the more complex needs of people living with HIV, as outlined in Table 1.

**TABLE 1:** Differences and similarities in polypharmacy research in older populations versus older HIV populations.

Aspect	Older populations	Older populations with HIV
Research focus	Preventing inappropriate prescribing Deprescribing Minimizing adverse events	Managing drug–drug interactions ART adherence Long‐term effects of lifelong treatment
Primary drivers	Age‐related co‐morbidities Physiological decline Frailty Inappropriate prescribing	Age‐related co‐morbidities Physiological decline Frailty Inappropriate prescribing HIV‐mediated effects Long‐term antiretroviral therapy (ART) use
Onset	Accumulates gradually with age and multimorbidity Typically, later in life (≥50 years)	May begin earlier due to HIV‐related co‐morbidities and ART initiation Middle‐aged (40–60 years), a decade earlier than people without HIV
Medication composition	Non‐HIV medications	Non‐HIV medications ART (typically three or more medications)
Adverse outcomes	Hospitalizations Mortality Functional decline Reduced quality of life Prescribing cascade Cognitive impairment Falls and fractures	Similar outcomes to older populations without HIV, in addition to: ART non‐adherence Drug–drug interactions between ART and non‐ART drugs
Beneficial outcomes	Improved management of multiple chronic conditions Symptom control and better quality of life Prevention of complications and hospitalizations Adherence to guideline‐recommended therapies	Similar benefits to older populations without HIV, in addition to: Maintenance of viral suppression through ART adherence Prevention of HIV‐related complications
Guideline availability	Beers Criteria STOPP/START STOPPFrail	Lack of standardized tools available

Polypharmacy can be considered universal in people with HIV when ART is included, since ART, typically consisting of two or three drugs, is offered to all individuals, resulting in what might be referred to as “premature” polypharmacy [9]. Data from the North American AIDS Cohort Collaboration on Research and Design showed that two‐thirds of eligible individuals initiating ART were in their 30s or 40s, with 17% under 30 years of age [10]. This suggests that people with HIV are exposed to polypharmacy decades earlier than people without HIV, who typically start medication for chronic conditions in their 50s or later. This earlier onset is compounded by HIV‐related factors, including chronic inflammation, ART‐related toxicities and altered immune recovery, that can contribute to the development of co‐morbidities such as cardiovascular disease (CVD), osteoporosis, renal impairment and cognitive disorders [11, 12]. Current HIV guidelines, including those from the British HIV Association (BHIVA) and European AIDS Clinical Society (EACS), also recommend earlier treatment initiation for certain co‐morbidities [4]. For example, BHIVA advises offering statins to all people with HIV aged 40 years and older for primary prevention of CVD, regardless of lipid profile or estimated CVD risk [13]. EACS recommends statins for those with a 10‐year CVD risk of ≥5% or higher, or even ≥2.5% for those under 50 years of age (i.e., moderate‐intensity statin if risk ≥2.5%) [14]. Although many of these prescribing decisions are beneficial for preventing and managing co‐morbidities, they increase medication burden and raise concerns that the overall regimen, due to the cumulative effects of multiple drugs, may become counterproductive.

Multiple cohort studies consistently report a high prevalence of polypharmacy in people with HIV^2^, particularly among those with CVD and mental health disorders [15]. Additional risk factors include older age, female sex and socioeconomic disadvantage [4, 16]. As this population ages, physiological changes, including increased frailty and altered drug metabolism, can also increase the risks of inappropriate prescribing and adverse drug effects [17, 18]. Polypharmacy in people with HIV has been linked to increased risks of drug–drug interactions (DDIs) [19], reduced ART adherence [20] and health‐related quality of life (even with suppressed viral loads) [15], and higher healthcare costs due to the complexity of managing DDIs [21].

Despite its clinical relevance, there is no universally accepted definition of polypharmacy in people with HIV. Most studies continue to use a threshold of five or more medications, a definition that was initially established based on its ability to best reflect the risk of adverse health outcomes [22]. However, this may not adequately capture the unique circumstances of people with HIV, where long‐term exposure to ART can predispose individuals to adverse outcomes [23]. Moreover, the debate over whether ART should be included in polypharmacy definitions reflects a broader issue; medication counts alone may not adequately capture the appropriateness, risk profile or clinical relevance of a person's overall regimen. For example, individuals just below the threshold may still face significant risks of adverse drug interactions and suboptimal treatment outcomes, while those above thresholds may be considered to be overtreated despite being stable on multiple appropriate medications. This raises important questions about how best to define and assess polypharmacy in HIV care. Should definitions aim to quantify treatment burden, predict harm or guide deprescribing? Should they be standardized for research or flexible to adapt to individual clinical contexts? And how can we distinguish between necessary and excessive prescribing?

This review provides a comprehensive evaluation of polypharmacy research in the context of HIV. First, we examine how polypharmacy has been defined in existing HIV studies, and argue for a shift from purely quantitative thresholds to more nuanced, context‐sensitive definitions. Second, we provide an overview of clinical and structural predictors, as well as outcomes associated with polypharmacy. Third, we review the challenges of managing polypharmacy in clinical practice, with a focus on distinguishing appropriate from inappropriate prescribing and applying person‐centred strategies, such as deprescribing. By integrating these perspectives, we provide a framework to guide both the study and the management of polypharmacy in people living with HIV.

## HOW IS POLYPHARMACY DEFINED IN EXISTING HIV RESEARCH?

In populations without HIV, no standard definition for polypharmacy exists [24, 25]. One review identified over 140 different definitions, most of which rely solely on the number of medications taken with thresholds ranging from 2 to over 11 drugs [25]. To understand how polypharmacy is defined in people with HIV, we conducted a targeted literature search in March 2025 using MEDLINE, Web of Science and Scopus. We included original English‐language articles that defined polypharmacy in adults with HIV (aged ≥18 years) and specified which medications were considered. After screening 819 articles and removing duplicates, 37 studies met the inclusion criteria and are summarized in Table 2. Similar to other studies, definitions varied widely across HIV studies. Most studies used a numerical threshold, typically 5 or more medications, though some ranged from ≥4 to ≥11. Higher thresholds, such as those based on ≥10 or ≥11 medications, are often used to define “hyperpolypharmacy” or “severe polypharmacy.” Most studies also assess polypharmacy independently of diagnosed co‐morbidities. Few studies used alternative definitions, such as categorizing polypharmacy using ordinal groupings (e.g., none/low: 0–4 medications; moderate: 5–9; severe: ≥10), or based on the number of medication classes or co‐morbidities treated.

**TABLE 2:** Definitions of polypharmacy used in existing HIV studies.

Characteristic of definition	Number of studies (%)
Simple threshold count	37 (100)
Threshold used (number of drugs received)	
>4	1 (2.7)
>5	32 (86.5)
>6	3 (8.1)
Multiple thresholds	1 (27)
Included ART	
Yes	7 (18.9)
No	29 (78.4)
Not stated	1 (2.7)
Medication classes stated	
Yes	15 (40.5)
No	22 (59.5)
Classification tool used	
WHO ATC	11 (29.7)
ICD‐9‐CM	2 (5.4)
Korea Food and Drug Safety Ministry	1 (2.7)
Therapeutical indication class	1 (2.7)
Not stated	22 (59.5)
Included duration of medication	9 (24.3)
Prescribed medications only (excluding over‐the‐counter, herbal and supplements)	10 (27.0)

Abbreviations: ICD‐9‐CM, International classification of diseases, 9th revision, clinical modification; WHO ATC, World Health Organization anatomical therapeutic chemical classification.

Most studies exclude ART to focus on non‐HIV‐related medication burden. However, some incorporate both ART and non‐ART drugs, particularly when examining pill burden or DDIs, typically using a threshold of more than five or six medications. Few studies justified the exclusion of ART; in those that do, the rationale was to facilitate comparisons with people without HIV. Inconsistencies also arise in how multi‐drug formulations, including ART regimens, are counted, with some studies treating them as a single entity and others counting each active agent separately.

The criteria for medication inclusion also varied considerably. Many studies excluded self‐prescribed, herbal and recreational drugs, while others included over‐the‐counter medications (OTC) and supplements if documented in medical records or self‐reported during questionnaires/surveys at study visits. Given that medication use may change over time, it is important to consider administering these questionnaires at multiple time points rather than relying solely on a single assessment, which may not accurately capture ongoing or intermittent medication use. Medication duration is another area of inconsistency. Most studies define polypharmacy based on medications prescribed for at least 3–6 months, while others take a broader approach, including medications prescribed at any point during the study period. The range of medication classes considered also differed across studies, with less than half specifying which classes were included. Some referenced classification systems (e.g., ICD‐9‐CM and WHO Anatomical Therapeutic Chemical (ATC) classification system) but did not list the specific classes examined. Among studies that did, the number of included classes ranged from six to 30, with the WHO ATC being the most commonly used system. However, most studies did not explain the rationale for their selection. Cardiovascular medications (e.g., antihypertensives, statins and anticoagulants) and central nervous system agents (e.g., antidepressants, antipsychotics and anticonvulsants) were the most frequently included drug classes. Other commonly considered categories included gastrointestinal agents (e.g., proton pump inhibitors and drugs for acid‐related disorders), analgesics, and anti‐infectives (e.g., antivirals, antibacterials and antifungals). In contrast, dermatological agents, sensory organ medications (e.g., ophthalmologic drugs), vitamins and herbal supplements were less frequently considered, despite their potential contributions to DDIs and adverse drug reactions.

## PREDICTORS OF POLYPHARMACY

Older age has been consistently associated with polypharmacy [26]. Some studies suggest that longer duration of HIV infection, which is closely linked to older age, may also contribute to this association [27]. This relationship may reflect prolonged periods of viral replication and cumulative exposure to older ART regimens, which have been implicated in cellular senescence [28]. In studies of middle‐aged and older adults with HIV, female sex has also been associated with higher rates of polypharmacy. This may partly reflect the use of hormonal treatments (e.g., HRT and contraceptive medications) [29, 30], but other factors, such as differences in comorbidity patterns and healthcare engagement, are also likely to play a role [30, 31, 32, 33]. Females are more likely to have frequent contact with healthcare services, increasing opportunities for disease detection and prescribing, and more likely to seek preventive care compared with males. Clinical factors including frailty, recent hospitalizations, lower nadir CD4 counts, and co‐morbidities, particularly cardiovascular and neurological conditions, have also been independently linked to increased medication burden [34, 35]. Additionally, treatment‐related factors, such as longer ART duration (which reflects prolonged HIV infection) and the use of integrase inhibitors (INSTIs), have been associated with a higher odds of polypharmacy [34]. This may partly reflect clinical practice where individuals on multiple medications are switched from boosted protease inhibitors (PIs) to INSTIs to reduce drug–drug interactions.

## ASSOCIATIONS WITH HEALTH OUTCOMES

Several large observational studies have shown that a higher number of non‐antiretroviral (ARV) medications is independently associated with adverse health outcomes in people with HIV. However, causality is difficult to establish, as many outcomes may also stem from underlying co‐morbidities. One of the most consistent associations is with falls and fractures [36, 37, 38]. Data from the Veterans Aging Cohort Study (VACS) showed that every five additional non‐ARV medications increased serious fall risk by 19%, even after adjusting for age, comorbidity burden and functional status [37]. Specific medication classes, including benzodiazepines, anticonvulsants, muscle relaxants and antiarrhythmics, were found to be strongly associated with increased risk of serious falls. This is particularly concerning in people with HIV, who are at a higher risk of low bone mineral density [39]. Polypharmacy has also been linked to functional decline, a concern that is already well recognized in this population due to underlying factors like chronic inflammation and cognitive impairment. Among middle‐aged and older people with HIV, higher medication burden is associated with frailty, reduced physical function, and performance on gait speed and grip strength [35, 40, 41]. Furthermore, severe polypharmacy (defined as ≥10 non‐ART medications) has been linked to poorer cognitive outcomes, including reduced executive function and psychomotor speed, especially in older women with HIV aged 50 years or older who are virally suppressed [42].

Some studies have also shown that polypharmacy predicts both hospitalization and all‐cause mortality [4]. Using data from VACS, one study reported a 50% higher risk of hospitalization and a 43% higher risk of death among people with HIV prescribed multiple non‐ARV medications, even after adjusting for age, sex, race/ethnicity and VACS Index [43]. These findings argue against residual confounding as the sole explanation for these associations, instead supporting the hypothesis that polypharmacy itself may be a modifiable contributor to poor health outcomes. Additionally, polypharmacy has been independently associated with non‐adherence to ART [20, 44]. However, it is important to consider the multifactorial nature of adherence, which is influenced not only by increased pill burden but also by behavioural, social and psychological factors, such as stigma, lack of confidence in immediate or future benefits and concern about DDIs [45, 46]. For instance, qualitative findings from the 2019 Positive Perspectives survey showed that higher medication burden is linked to lower satisfaction with treatment, greater concern about risks of DDIs and poorer perceived health status [15]. Finally, polypharmacy has been associated with an increased risk of DDIs and prescribing challenges, including inappropriate medication use, prescribing cascades and interactions with co‐morbidities [43, 47, 48].

## DEFINING POLYPHARMACY IN HIV RESEARCH: CONSIDERATIONS AND METHODOLOGICAL CHALLENGES

The term “polypharmacy” is often used with assumptions, such as a universal threshold of five or more medications, equal risk contribution from all medications, or that taking multiple medications is inherently detrimental. Grounded in existing literature, this section presents our perspective on key methodological considerations for defining polypharmacy in HIV research.

**FIGURE 1:**
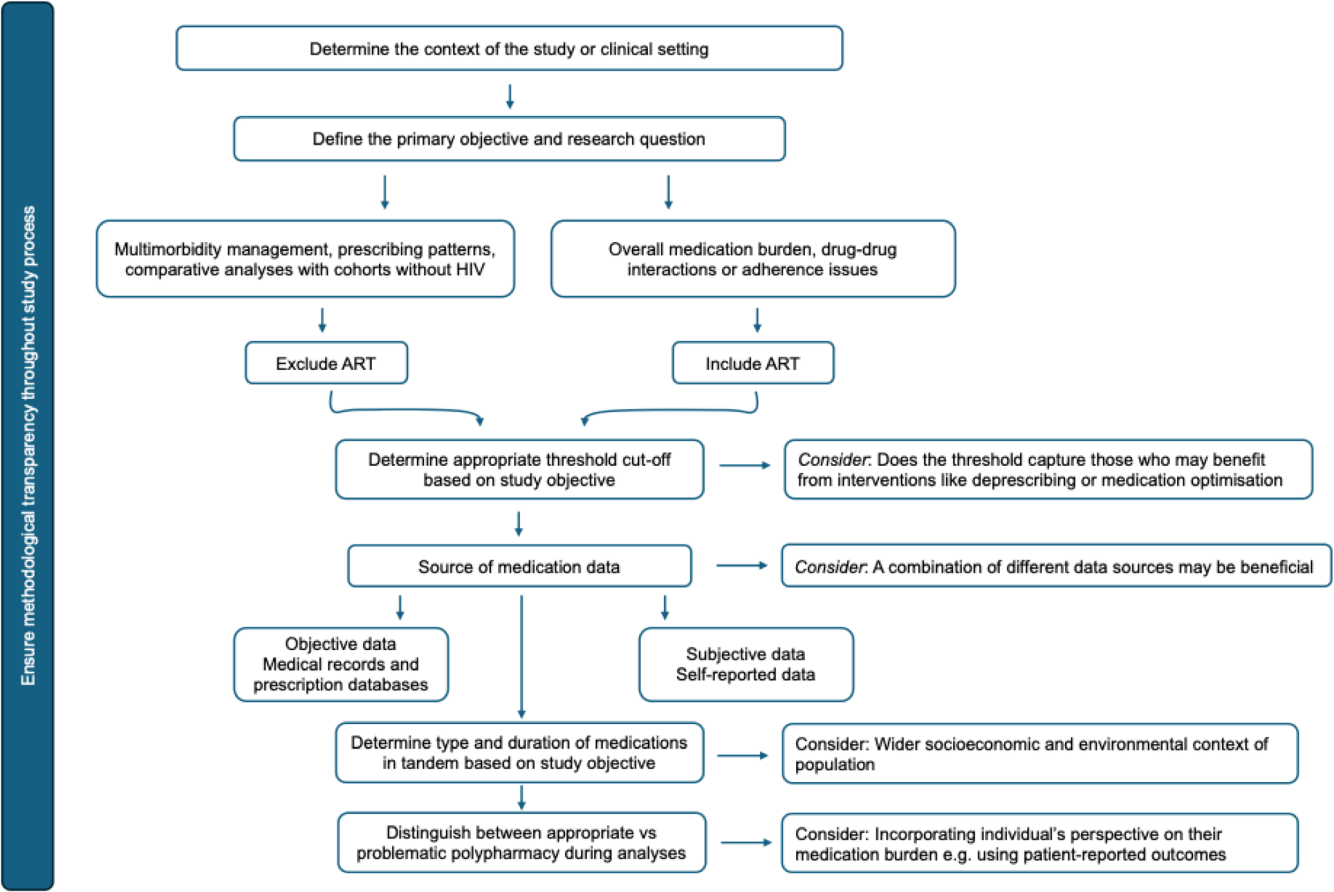
Framework to help researchers select or construct appropriate definitions for polypharmacy in people living with HIV.

We propose a decision‐support framework (see Figure 1) to guide researchers in selecting definitions that are tailored to their study objectives. ART may be included in analyses of multimorbidity and prescribing patterns, particularly when assessing overall medication burden, DDIs or adherence issues, as it contributes to regimen complexity and may necessitate additional treatments. Conversely, excluding ART may be appropriate in studies focusing on non‐HIV medications to identify potentially modifiable prescribing patterns or to facilitate comparisons with non‐HIV populations. Both approaches are valid when clearly justified, with decisions guided by the research question and broader therapeutic context.

Several methodological factors shape how polypharmacy is defined in HIV research. For example, the selection of an appropriate threshold for medication count is important. While five or more medications are commonly used, this threshold may not fit all research contexts. Lower thresholds can increase sensitivity, capturing more individuals at risk of adverse effects, but may overestimate polypharmacy burden. For instance, individuals who take multiple medications for conditions unrelated to HIV (such as hypertension or diabetes) may be classified as experiencing polypharmacy, even if their regimen does not significantly impact their health or HIV treatment outcomes. Conversely, higher thresholds reduce sensitivity and risk missing individuals who experience significant medication burden but fall below the cutoff. Therefore, the threshold selected should align with the study's purpose, whether identifying individuals for interventions like deprescribing or assessing the risk of adverse outcomes, and be reported transparently along with contextual factors, such as how medication burden is measured (drug count, pill count, dosing frequency), study population characteristics and the primary research question. Data sources can also impact how polypharmacy is defined. Medical records and prescription databases provide objective, clinically verified data but may miss medications obtained outside formal healthcare settings. Self‐reported data can provide a more comprehensive picture of an individual's full medication regimen, but is more susceptible to recall bias. Where possible, using multiple data sources can help mitigate these biases and provide more robust estimates of polypharmacy.

Another important methodological consideration is the type and duration of medications. Prescribing patterns in people with HIV may shape the types of risks observed. For example, medications commonly used to manage or prevent medical conditions closely linked with HIV (such as statins) contribute differently to overall medication burden than medications prescribed for conditions with less clear linkage to HIV (such as antihypertensives). The inclusion of OTC medications, herbal remedies and supplements in polypharmacy definitions also remains contentious. Their inclusion could offer a more holistic view of medication burden, especially in older adults or those self‐managing symptoms for, say, insomnia or gastrointestinal discomfort. While not all OTC medications exert clinically meaningful effects, overlooking these agents may lead to underestimating adherence barriers, unmonitored drug interactions with ART or missing adverse effects [49, 50]. Another source of variability stems from whether definitions incorporate duration, such as requiring medications to be taken for a minimum number of months. While this can distinguish chronic from transient use, it may exclude short‐term regimens that still pose risks. For instance, an individual taking nine medications for 2 months could be dismissed as not meeting the criteria for polypharmacy, while someone on five medications for 4 months would qualify, despite both potentially facing similar risks of DDIs, adverse drug reactions or adherence barriers. Therefore, definitions should consider both duration and intensity of exposure, with flexibility to also account for the presence and severity of adverse effects.

Contextual and structural factors can also influence how polypharmacy is experienced and studied in HIV populations. Socioeconomic status (SES), healthcare access, medication affordability and health literacy can influence medication use and adherence [51]. Individuals from lower socioeconomic backgrounds may face challenges with adhering to complex medication regimens as well as accessing or affording these medications, particularly in settings without insurance coverage. While evidence in populations without HIV suggests higher odds of polypharmacy among those with lower SES [52], the interplay of social factors in people with HIV remains underexplored. Patient‐reported outcomes [53] may provide an additional lens for understanding polypharmacy beyond numerical counts. Measures such as perceived medication burden, treatment satisfaction, and medication‐related side effects may not correlate directly with medication count, but can reveal important aspects of adherence, quality of life and clinical outcomes. We propose that quantitative measures should be complemented by assessments of contextual and structural factors. This would ensure that polypharmacy is not just viewed from a clinical or quantitative standpoint, but also from the lived experience of individuals with HIV.

Taken together, these definitional and methodological considerations provide a foundation for understanding the complexities of managing polypharmacy in people living with HIV. In the following section, we review the current evidence on clinical challenges, including regimen complexity, DDIs and deprescribing.

## CURRENT CHALLENGES IN THE MANAGEMENT OF POLYPHARMACY IN HIV

Polypharmacy in older people with HIV is often viewed through a stigmatizing lens, with attention focused almost exclusively on negative outcomes such as medication overload, suboptimal care and reduced quality of life. This perspective overlooks the clinical realities of ageing with HIV. As people with HIV age, they develop co‐morbidities earlier and more frequently than their HIV‐negative counterparts, often requiring complex medication regimens that may be aligned with evidence‐based guidelines. In such cases, polypharmacy may be appropriate and necessary. Nevertheless, the potential for harm increases when medication regimens become excessive, misaligned with clinical goals, or poorly coordinated across healthcare providers. Thus, polypharmacy should be evaluated based on appropriateness rather than simply the number of medications. Appropriate polypharmacy follows clinical guidelines and meets the individual's therapeutic needs, whereas inappropriate polypharmacy involves unnecessary, potentially harmful, or duplicative medications. Instead of automatically viewing polypharmacy as problematic or indicative of poor care, it should prompt careful, person‐centred evaluation to ensure that each medication remains appropriate, safe and aligned with the individual's evolving health needs.

Older adults with HIV are particularly vulnerable to inappropriate prescribing due to age‐related physiological changes, altered pharmacokinetics, and a high burden of multimorbiditiy [32]. This increases the potential for both recognized and unrecognized drug–drug and drug–disease interactions [54]. Common examples include corticosteroids destabilizing glycaemic control in those with diabetes [55], or tenofovir disoproxil fumarate exacerbating existing bone or renal disease [56]. Inappropriate prescribing can involve potentially inappropriate medications (PIMs), where the potential for harm outweighs clinical benefit, or prescribing cascades, where an adverse event of one medication is misinterpreted as a new clinical diagnosis and treated with another drug, putting the individual at further risk of adverse reactions [4]. Studies indicate that 52%–69% of older people with HIV are prescribed PIMs, primarily from non‐ART drugs [47, 57]. Other common prescribing issues include drugs prescribed and carried on even though there is no clinical indication or dosing errors (e.g., drugs not adjusted to the renal function of the patient). The EACS guidelines provide a table for non‐HIV drugs predominantly eliminated renally which require dose adjustment in case of renal impairment.

In the absence of HIV‐specific prescribing tools, clinicians often rely on tools developed for elderly populations without HIV. These fall into two main categories: explicit and implicit tools. Explicit tools, such as the Beers criteria [8] and the STOPP/START, provide standardized lists of PIMs, based on literature reviews and/or expert consensus. While implicit tools such as the medication appropriateness index (MAI) [58] offer more nuanced, person‐specific assessments, considering an individual's overall health status, treatment goals, and medication burden, they are time‐consuming and resource‐intensive, requiring expert input from clinical pharmacists, geriatricians and/or HIV specialists. A recent scoping review underscored the high prevalence of specific PIMs identified using these tools in people with HIV [59]. Benzodiazepines are among the most frequently identified PIMs, and their long‐term use is particularly concerning given their well‐documented associations with neurocognitive impairment, reduced structural brain integrity, and serious falls [37, 60, 61]. Benzodiazepine is more commonly prescribed in HIV populations [62], likely due to higher rates of anxiety disorder, sleep disturbances and current substance use [63, 64, 65]. These medications are also sometimes initiated to manage symptoms related to stimulant use or prescribed for prolonged periods in individuals with past addictions. Despite their widespread use, benzodiazepines are recognized as a key target for de‐prescribing efforts [66], although withdrawal strategies can be complex. Other common PIMs include prolonged use of nonsteroidal anti‐inflammatory drugs (NSAIDs), antidepressants, opioids and medications with high anticholinergic burden [67, 68]. Importantly, underprescribing, or “potential prescribing omissions”, of beneficial treatments has also been reported. For instance, START criteria revealed missed opportunities for initiating preventive therapies such as calcium and vitamin D supplementation for bone health and fracture prevention. This highlights the limitations of applying general tools to HIV populations with distinct clinical needs, and the importance of developing condition‐specific indicators.

The majority of the existing literature focuses on explicit tools, though some studies also assess implicit approaches, often in combination with explicit criteria. Clinical pharmacists have been key in these initiatives, using structured medication reviews, adherence‐promoting tools and collaborative care models to effectively target individuals with high medication burden (typically ≥11 chronic medications) for comprehensive review and deprescribing [57]. Multidisciplinary collaborations between HIV specialists and geriatricians have also shown promise to improve prescribing safety. Individual‐facing tools, such as the management optimization review (MOR), have empowered individuals to review their own medications and identify potential concerns in partnership with clinicians [69].

Deprescribing, the systematic discontinuation of medications when harms outweigh benefits, is increasingly recognized as a feasible and safe practice in populations without HIV [70]. Two main strategies exist: “cutting” (systematically discontinuing PIMs using explicit tools) and “pruning back” (a more individualized, context‐specific process) [71]. The former is simpler to implement, but the latter better aligns with person‐centred care. HIV‐specific deprescribing resources are emerging. The European AIDS Clinical Society (EACS) has published targeted de‐prescribing lists (e.g., “Top 10 Drug Classes to Avoid in Elderly People Living with HIV,” and “Drug Classes to Deprescribe in Older Persons with HIV in Presence of Certain Conditions”) [66, 72]. More systematically, the Spanish AIDS Study Group (GESIDA) has developed a de‐prescribing guide, which includes decision algorithms tailored to commonly used drug classes like benzodiazepines and antipsychotics. This guide also provides clinical risk thresholds to identify candidates for de‐prescribing, including individuals aged 50 years or over, those taking ≥5 chronic medications and those with a high veterans aging cohort study (VACS) index score [73, 74]. This represents a step toward operationalizing de‐prescribing within HIV care, though real‐world implementation data remain sparse, and its applicability across diverse healthcare systems is yet to be established. Currently, it is unclear whether the evolution of ART itself may also present opportunities to reduce medication burden. Emerging long‐acting injectable therapies, such as cabotegravir and rilpivirine, may simplify ART regimens by allowing infrequent dosing due to their prolonged half‐lives, potentially improving adherence and treatment satisfaction. Although these agents have a low potential to cause DDIs themselves, their effectiveness can still be influenced by concomitant medications that alter their metabolism, so DDI risks are not completely eliminated [75, 76]. Their prolonged release and slow clearance can also create an extended window during which interactions with other medications can occur [77]. As these drugs cannot be rapidly reversed or removed, managing interactions and adverse events becomes more complex. Beyond ART, polypharmacy increasingly includes other long‐acting therapies, such as hormonal contraceptives, osteoporosis therapies and antipsychotics, further complicating dosing, monitoring and management of interactions. Furthermore, the long‐term safety and efficacy of newer regimens in older or frail populations with HIV remain under‐evaluated. Many older individuals with HIV have complex ART histories, prior resistance mutations or residual immunosuppression, factors that may limit their eligibility for newer treatment approaches [73]. Additionally, frailty, cognitive impairment and challenges with clinic attendance may further complicate their use. In certain frailty or end‐of‐life contexts, studies should explore ethical and clinical frameworks for ART de‐intensification. Several practical and systemic challenges should also be considered. These include the lack of standardized HIV‐specific guidelines, fear of destabilising well‐controlled conditions or triggering withdrawal effects, and residual uncertainty or mistrust from past toxic ART exposure (also referred to as ‘pharmacological trauma’). Polypharmacy often also results from multiple prescribers/specialties, making medication reconciliation and coordinated de‐prescribing plans more difficult. Medication transparency may also be compromised by stigma, particularly around HIV and/or mental health, which may hinder honest disclosure of nonadherence or non‐prescribed medication use. Finally, although subjective experience and perceptions of medication burden can vary between individuals, deprescribing strategies often fail to incorporate individuals' preferences or encourage shared decision‐making.

## RECOMMENDATIONS FOR CLINICAL PRACTICE AND RESEARCH

**FIGURE 2:**
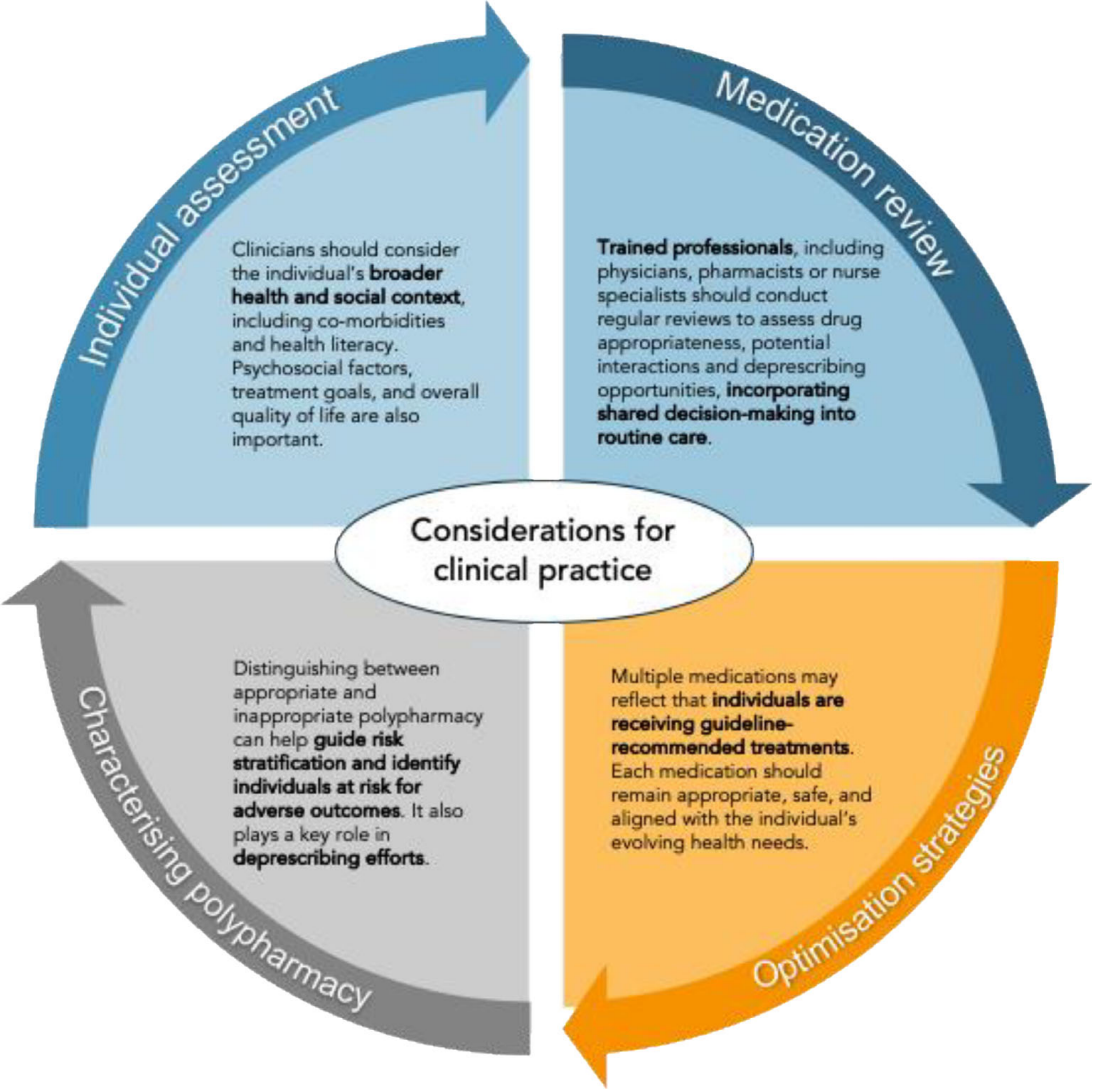
A person‐centred framework for the management of polypharmacy in people living with HIV. This framework builds on geriatric principles of medication optimization but is adapted to the unique needs of HIV populations.

Building on the definitional and clinical challenges outlined above, we propose a practical, HIV‐specific framework (Figure 2) built on geriatric principles of medication optimization and adapted to reflect the needs of HIV populations with polypharmacy. Applying this framework to distinguish between inappropriate and appropriate polypharmacy not only ensures clinical safety but also helps avoid stigmatizing patients with complex, guideline‐concordant regimens. This approach recognizes that multiple medications are not inherently problematic when clinically justified.

The first step involves a comprehensive medication reconciliation and review, which should be conducted by trained professionals, such as physicians, clinical pharmacists or nurse specialists, with relevant expertise to assess drug appropriateness, potential interactions and de‐prescribing opportunities. This critical, though time‐intensive process should be performed annually (or more frequently if feasible) and updated at every visit. Efforts should be made to simplify the number of prescribers involved in an individual's care, which can reduce conflicting recommendations and fragmented treatment plans. Encouraging individuals with HIV to bring all their medications to appointments (known as the “brown bag” review method [78]), including prescription drugs, OTCs, vitamins and herbal remedies, can allow clinicians to identify duplications, inappropriate combinations and undisclosed therapies. Clinicians should also engage in open discussions with individuals with HIV about their adherence levels and any associated symptoms. Exploring barriers to adherence, such as forgetting doses, perceived ineffectiveness, difficulty taking pills or cost, is important for developing targeted solutions. At this stage, the involvement of clinical pharmacists or HIV‐specialist pharmacists may be important to differentiate between appropriate and inappropriate polypharmacy. Their expertise can help flag inappropriate combinations, adjust doses based on pharmacokinetic changes, and identify any harmful interactions with ART. The second step involves assessing medications and prioritizing them based on their benefits and risks using standardized tools, clinical judgement, and HIV literature. ART remains the cornerstone of HIV treatment and is non‐negotiable. As a core component of polypharmacy, ART contributes to medication complexity and potential interactions, and decisions about ART regimen selection, including triple therapy, two‐drug combinations or long‐acting injectables, should always be considered in the context of the individual's total medication burden. Polypharmacy management strategies should therefore prioritize maintaining ART adherence and efficacy while carefully assessing interactions between ART and non‐ART medications, particularly for regimens involving PIs or cobicistat‐boosted agents, as well as between non‐ART drugs, which are increasingly common in people with HIV [19]. Special attention should also be paid to known high‐risk drug classes in people with HIV, such as proton pump inhibitors linked to fracture risk [79] or opioids associated with immune suppression, dependence and adverse effects [80, 81]. In some instances, behavioural and non‐pharmacological alternatives, such as cognitive behavioural therapy and counselling, may be an appropriate alternative [82]. Third, people with HIV frequently experience a higher burden of mental disorders, substance use, social instability and stigma, all of which influence medication adherence, access to care and tolerance to medication side effects [83]. These psychosocial factors, often more prevalent in people with HIV than geriatric populations, must be integrated into medication decision‐making. Clinicians should also promote shared decision‐making by including individuals with HIV to discuss their preferences, values, and health literacy. For instance, engaging them in discussions and jointly ranking medications by perceived benefit or burden can guide de‐prescribing discussions and enhance adherence to high‐priority medications [84]. This collaborative approach also ensures that de‐prescribing targets inappropriate polypharmacy while safeguarding necessary, guideline‐concordant treatments. Finally, studies suggest that people with HIV may exhibit features of premature biological ageing [85], requiring personalized assessments of physiological reserve and treatment tolerance rather than relying solely on chronological age cutoffs. Tools used in geriatrics should be adapted to account for this.

**FIGURE 3:**
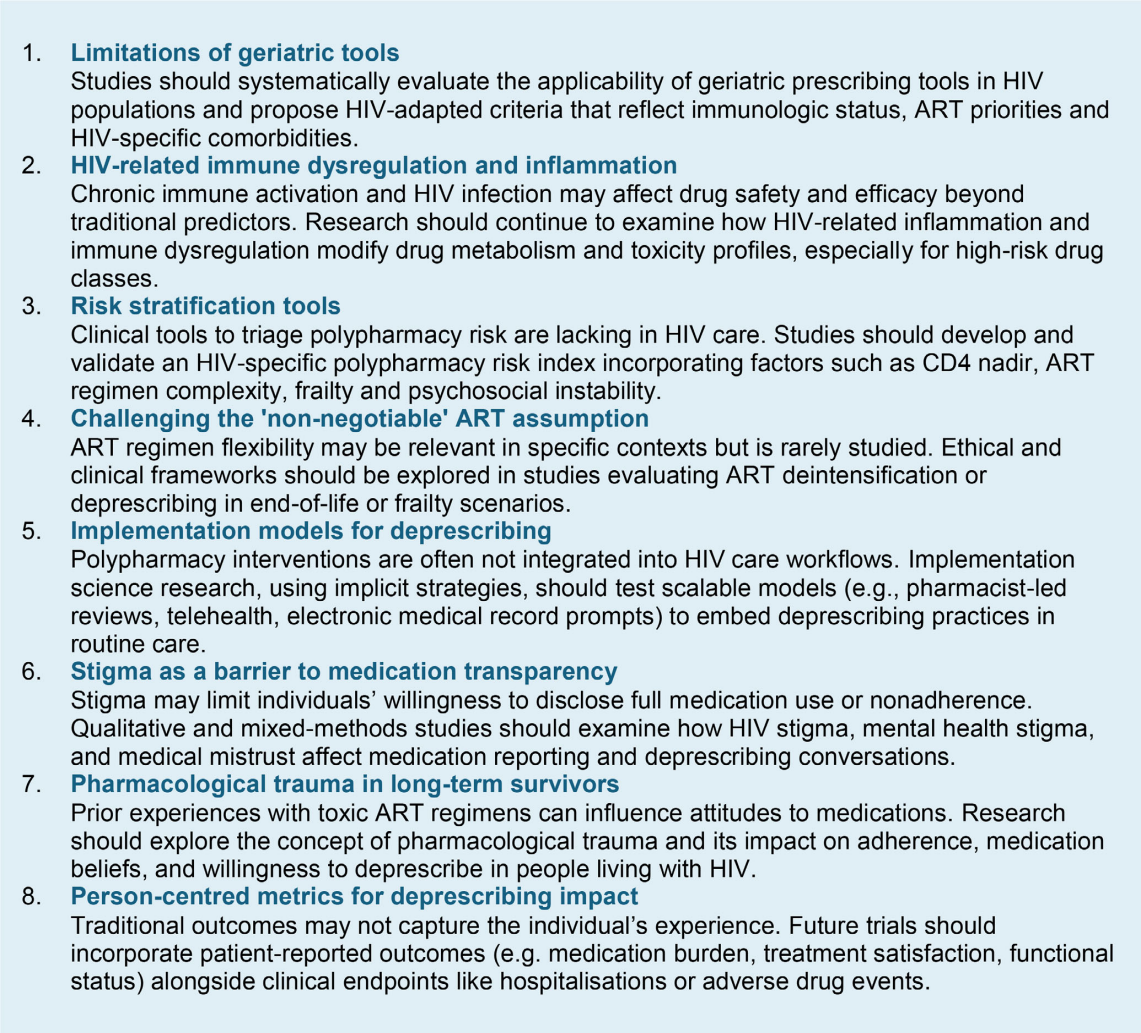
Priorities for future research and practice.

Given the absence of HIV‐specific guidelines, there is a pressing need for consensus‐driven recommendations developed through interdisciplinary collaboration. Initiatives such as the HIV and Aging Consensus Project represent promising steps forward, who recommend treatment strategies for clinicians who manage older adults with HIV [86], but broader uptake and international adaptation are needed. Expert panels comprising clinicians, pharmacists, researchers and community representatives should review existing evidence to refine definitions of inappropriate polypharmacy, establish criteria for PIMs in people with HIV, and provide guidance for maintaining appropriate polypharmacy. However, the development of guidelines must be supported by robust, evidence‐based research. Medication review remains a time‐consuming process, especially in groups with complex treatment patterns such as older adults with HIV. Artificial intelligence (AI) is emerging as a promising tool to support prescribers by streamlining medication review and identifying potentially inappropriate prescriptions, with early research efforts already underway to implement such technologies [87]. At present, it remains unclear which interventions are most effective in reducing inappropriate polypharmacy in people with HIV, whether adapted geriatric tools improve prescribing quality, and what their downstream effects are on clinical outcomes. Future research, including rigorously designed real‐world studies that address potential biases, is essential to answer these questions. Understanding what works and what does not will be crucial to developing meaningful, scalable strategies for safer and more effective medication use in the ageing HIV population. To support such initiatives, Figure 3 outlines key areas of unmet need in polypharmacy research in the context of HIV.

## CONCLUSION

In HIV care, there is a need to refine how polypharmacy is defined in research and to re‐evaluate how it is managed in practice. Definitions must move beyond simple medication counts to capture appropriateness, risk profile and patient‐reported burden. At the same time, management requires a person‐centred approach that distinguishes appropriate, which is evidence‐based and clinically necessary, from inappropriate polypharmacy, which may pose harm or provide limited benefit. Tools developed for elderly populations without HIV offer useful starting points, but their limitations highlight the need for HIV‐specific frameworks that address the unique clinical and psychosocial complexities of this group. Integrating deprescribing into routine HIV care, through multidisciplinary teams, structured medication reviews and shared decision‐making, may support safer, more appropriate prescribing in this population. Future research and interdisciplinary collaboration are crucial to refining proposed definitions and developing consensus standards that will improve the management of polypharmacy among people living with HIV.

## AUTHOR CONTRIBUTIONS

The work reported in this review was designed and conducted by all authors. LS and CS conceptualised the piece, and LS drafted the initial version based on written and oral inputs from all authors. LS conducted the literature review. All authors reviewed the drafts and provided substantive feedback, with further revisions led by LS. All authors reviewed and approved the final version prior to submission. All authors had full access to the resources, materials, and information used in developing this work.

## Data Availability

Data sharing not applicable to this article as no datasets were generated or analysed during the current study.
